# The Paradigm Change of IL-33 in Vascular Biology

**DOI:** 10.3390/ijms222413288

**Published:** 2021-12-10

**Authors:** Svitlana Demyanets, Stefan Stojkovic, Kurt Huber, Johann Wojta

**Affiliations:** 1 Department of Laboratory Medicine, Medical University of Vienna, 1090 Vienna, Austria; svitlana.demyanets@meduniwien.ac.at; 2 Department of Internal Medicine II, Division of Cardiology, Medical University of Vienna, 1090 Vienna, Austria; stefan.stojkovic@meduniwien.ac.at; 3 3rd Medical Department with Cardiology and Intensive Care Medicine, Clinic Ottakring, 1160 Vienna, Austria; kurt.huber@meduniwien.ac.at; 4 Medical School, Sigmund Freud University, 1020 Vienna, Austria; 5 Ludwig Boltzmann Institute for Cardiovascular Research, 1090 Vienna, Austria; 6 Core Facilities, Medical University of Vienna, 1090 Vienna, Austria

**Keywords:** interleukin-33, ST2, atherosclerosis, thrombosis, vascular

## Abstract

In this review, we focus on the actual understanding of the role of IL-33 in vascular biology in the context of the historical development since the description of IL-33 as a member of IL-1 superfamily and the ligand for ST2 receptor in 2005. We summarize recent data on the biology, structure and signaling of this dual-function factor with both nuclear and extracellular cytokine properties. We describe cellular sources of IL-33, particularly within vascular wall, changes in its expression in different cardio-vascular conditions and mechanisms of IL-33 release. Additionally, we summarize the regulators of IL-33 expression as well as the effects of IL-33 itself in cells of the vasculature and in monocytes/macrophages in vitro combined with the consequences of IL-33 modulation in models of vascular diseases in vivo. Described in murine atherosclerosis models as well as in macrophages as an atheroprotective cytokine, extracellular IL-33 induces proinflammatory, prothrombotic and proangiogenic activation of human endothelial cells, which are processes known to be involved in the development and progression of atherosclerosis. We, therefore, discuss that IL-33 can possess both protective and harmful effects in experimental models of vascular pathologies depending on experimental conditions, type and dose of administration or method of modulation.

## 1. Interleukin (IL)-33 Biology, Structure and Signaling

Identified by Schmitz et al. [1] in 2005 as a member of the IL-1 family of cytokines, IL-33 is recognized as a dual-function factor. Firstly, this is a nuclear-located factor with the ability to regulate transcription. Secondly, IL-33 acts as an extracellular cytokine upon its release from cells exposed to stress [2,3,4].

The IL-33 gene is located on chromosome 9p24.1 in humans, whereas in mice it was detected on the syntenic chromosome 19qC1 region. The comparison of human and mouse IL-33 showed 55% identity at the amino acid level [1]. Sequence-based homology and three-dimensional analysis confirmed the structural similarity of IL-33 to IL-1 and IL-18 [5,6]. However, Rivers-Auty et al., who investigated the evolutionary history of the IL-1 superfamily, concluded that IL-33 and IL-18 have no chromosomal evidence of common ancestry with the IL-1β cluster. For that reason, they advised using the term “IL-1 superfamily” instead of “IL-1 family” [7].

IL-33 contains seven coding exons, which produce a ~31 kilodalton (kDa) protein of 270 amino acids in humans and 266 amino acids in mice. The N-terminal domain (amino acids 1–65) required for IL-33 nuclear localization is encoded in exons 1–3. The C-terminal IL-1-like cytokine domain is encoded in exons 4–7 and is essential for binding to its receptor [8]. Structural analysis of IL-33 by multidimensional heteronuclear *NMR* spectroscopy revealed that IL-33 adopts a β-trefoil fold comparable to other IL-1 cytokines but with a unique insert of additional ten residues in the β4-β5 loop [9].

Suppression of tumorigenicity 2 (ST2; also known as IL1RL1/IL-1-receptor-like 1, DER4, Fit-1 or T1) was discovered more than 30 years ago as a serum-inducible secreted protein in murine fibroblast [10]. Being for a long time an orphan receptor, ST2 was finally identified as a part of the functional IL-33 receptor complex [1]. A further study demonstrated that a signaling receptor subunit IL-1R accessory protein (IL-1RAcP) is necessary for the signaling through ST2 [11]. ST2, but not IL-1RacP, possesses pronounced interdomain flexibility. Surface-charge complementarity at site 1 was defined to be the molecular mechanism by which ST2 specifically binds IL-33, and IL-33 acidic residues Glu148 and Asp149 at site 1 and Glu165 at site 2 are essential for high-affinity interaction with ST2 [6]. ST2 exists in different splicing variants based on the differential promoter binding, which results in either membrane-bound ST2 (ST2L), which acts as a receptor for IL-33 or soluble ST2 (sST2) [1,12]. Full-length ST2L contains an extracellular immunoglobulin (Ig)-like domain, a short extracellular spacer, the transmembrane domain and an intracellular toll/IL-1 receptor (TIR) domain [6]. All three Ig domains interact with IL-33. sST2 contains only the ectodomain of the receptor with three Ig fold domains but without the transmembrane helix. Therefore, sST2 is not attached to the plasma membrane and is a soluble secreted protein.

ST2 expressing cells react with the activation of nuclear factor-kappa B (NF-kB) and mitogen-activated protein kinases (MAPK) on stimulation by IL-33 with a preceding juxtaposition of the intracellular TIR domains of ST2 and IL-1RacP [1]. IL-33 also induces the recruitment of myeloid differentiation factor 88 (MyD88), IL-1R-associated kinase-1 (IRAK-1), IRAK-4 and tumor necrosis factor (TNF)-α receptor-associated factor 6 (TRAF6) to ST2L. Furthermore, the consequence of IL-33 stimulation was shown to be phosphorylation of inhibitor of κBα (IκBα), extracellular signal-regulated kinase1/2 (Erk1/2), p38, phosphoinositide-3-kinase (PI3K)/protein kinase B (AKT), janus kinase2 (JAK2), c-Jun N-terminal kinases (JNK) and spleen tyrosine kinase (SYK) [1,13]. Another study demonstrated the interaction of ST2L with transmembrane emp24 domain-containing protein 1 (TMED1), a member of the TMED/p24 family that is involved in the vesicular trafficking of proteins. Interestingly, this interaction augmented IL-33-induced IL-8 and IL-6 production [14]. In macrophages, IL-33 is involved in the regulation of phosphorylation of 672 proteins, among them the protein phosphatases protein tyrosine phosphatase non-receptor type 12 (PTPN12) and myosin phosphatase target subunit 1 (MYPT1) as well as activation of cdc42/Rho signaling involved in cell migration [15]. Pinto et al. reported phosphorylation of mitogen-activated protein kinase-activated protein kinase 2 (Mapkapk2), receptor-interacting protein kinase 1 (Ripk1), nicotinamide adenine dinucleotide (NAD) kinase and inositol polyphosphate-5-phosphatase D (Inpp5d) upon IL-33 stimulation [15]. The ST2/TRAF6/PI3K/Akt/endothelial nitric oxide synthase (eNOS) pathway is responsible for the production of NO and subsequent augmented vascular permeability and angiogenesis in human umbilical vein endothelial cells (HUVECs) [16].

Various modes of regulations of IL-33-dependent signaling are discussed. Main mechanisms include the neutralization of IL-33 by decoy receptor sST2, the phosphorylation of ST2L at S442 resulting in its internalization and further proteasome-mediated degradation and, finally, the inhibition of IL-33-dependet signaling via interaction of a TIR domain of single Ig IL-1R-related molecule (SIGIRR) with the IL-33 receptor complex [13,17,18]. Additionally, IL-33 activity can be regulated by alternate splice forms, microRNAs (miRs), oxidation of cysteine residues and proteolytic cleavage by proteases [13] as discussed in the next sections.

Therefore, IL-33 is a dual-function factor with both nuclear and extracellular cytokine properties, where it can activate multiple signaling pathways.

## 2. IL-33 as a Nuclear Factor

IL-33 was first described as a nuclear factor (NF) induced by subarachnoid hemorrhage (DVS27) in canine vasospastic brain arteries in 1999 [19] and further in 2003 [20] as NF from human high endothelial venules (NF-HEV) in secondary lymphoid tissue. NF-HEV is highly expressed in HEVECs compared to macrovascular HUVECs and nasal polyp-derived microvascular ECs. Baekkevold et al. concluded that NF-HEV, which contains a homeodomain-like helix-turn-helix (HTH) motif in its amino-terminal part, may represent one of the essential nuclear factors that regulate the HEV phenotype [20]. Further research described a homeodomain-like HTH motif within the IL-33 N-terminal part to be necessary for targeting IL-33 to mitotic chromosomes [8]. In parallel, Roussel et al. identified the docking of IL-33 into the acidic pocket formed by histone H2A–H2B dimer as the mechanism responsible for IL-33 binding to chromatin. This results in the regulation of chromatin compaction by encouraging nucleosome–nucleosome interactions. They also demonstrated the mimicry of the IL-33 chromatin-binding motif (CBM) by Kaposi sarcoma herpesvirus latency-associated nuclear antigen [21].

Two groups [22,23] demonstrated constitutive nuclear expression of IL-33 in ECs from both large and small blood vessels in normal human tissues from colon, small intestine, stomach, kidney, lung, liver, prostate, mammary gland and skin. In addition, IL-33 was shown to be constitutively expressed in the nucleus of epithelial cells in barrier tissues as well as of ECs from human tumors such as adenocarcinomas of the kidney, stomach, liver, pancreas, lung, breast or colon [22]. The investigation of inflamed tissues from patients with Crohn’s disease and rheumatoid arthritis revealed ECs as a major source of IL-33 mRNA also in chronically inflammation conditions [8]. Constitutive expression of nuclear IL-33 in blood vessels seems to be species-specific, as Pichery et al. detected no IL-33 expression in ECs along the vascular tree in normal mice [24]. That group revealed, similarly to previous reports in humans, abundant expression of IL-33 protein in mouse epithelial barrier tissues with nuclear but not cytoplasmic localization. In addition, endogenous IL-33 was detected in mouse lymphoid organs, brain and embryos by generation of Il-33–LacZ gene trap reporter strain [24].

In ECs, nuclear IL-33 is induced when cultured cells reach confluency and stop proliferating [23]. IL-33 was shown to be a marker of EC quiescence as cyclin-dependent kinase inhibitor p27 was expressed in almost all IL-33-positive cells. Moreover, Notch signaling is involved in regulating nuclear IL-33 expression in HUVECs in vitro and in vessels in vivo [25].

The definitive function of nuclear IL-33 is still not completely clear. Two reports [26,27] described an interaction of nuclear IL-33 with the transcription factor NF-κB with contradictory outcome effects. In the first report, Ali et al. revealed that full-length IL-33 interacts with NF-kB, which resulted in a reduction in NF-κB–mediated gene expression of IκBα, TNF-α and C-REL. IL-33 was shown to bind to the prototypical NF-κB p50 and p65 subunits with the biological effects observed upon the binding to p65. Contrarily, Choi et al. described that enhancement of NF-κB p65 subunits by ectopic overexpression of IL-33 in HUVECs results in the increased expression of intercellular adhesion molecule-1 (ICAM-1) and vascular cell adhesion molecule-1 (VCAM-1). At the same time, reduction in IL-33 by IL-33-targeting silencing RNA (siRNA) resulted in downregulation of NF-κB p65, adhesion molecules expression as well as monocyte adhesion to ECs in vitro. Knockdown of the ST2 receptor expression was performed in HUVECs prior to IL-33 overexpression in order to exclude the extracellular effects of IL-33. The authors postulated that, in this case, nuclear IL-33 exerted its activating effects on VCAM-1 and ICAM-1 in a receptor-independent manner in ECs [27]. A later publication by Shao et al. [28] showed again by a knockdown of IL-33 using siRNA in vitro that nuclear IL-33 regulates sST2 release and IL-6 expression in human arterial ECs.

IL-33 is known as a strong regulator of T helper type 2 (Th2 type) cytokines. Ni et al. demonstrated that nuclear IL-33 positively regulates the transcription of IL-13, one of the Th2 cytokines, by binding to the conserved non-coding sequence before the translation initiation site in the IL13 gene locus. Deubiquitination of IL-33 by the enzyme ubiquitin-specific protease 17 was revealed as the mechanism for the negative regulation of IL-33 nuclear function and stability [29]. Another study identified a member of the importin family of nuclear transport proteins, importin-5, as an intracellular binding partner of full-length IL-33 but not mature IL-33. The consequence of this interaction is the control of full-length IL-33 intracellular degradation [30].

However, several later reports detected no evidence for a role of nuclear IL-33 in regulating gene expression [31,32]. In primary human ECs, knockdown of endogenous nuclear IL-33 expression by two different strategies based on siRNA pools had no substantial effect on the proteome. This study concluded that the main purpose of IL-33 nuclear localization seems to be the regulation of its extracellular cytokine activity through nuclear sequestration [32].

Therefore, IL-33 is an endothelium-derived, heterochromatin-associated nuclear factor, which may process transcriptional repressor properties under certain circumstances. However, the definitive role of nuclear IL-33 is still not clarified.

## 3. Cellular Sources and Expression under Pathological Conditions and Mechanisms of IL-33 Release

IL-33 is broadly expressed in different cell types, but shows a diverse expression pattern in comparison to IL-1β and IL-18. Although hematopoietic cells seem to be the main source of IL-1β and IL-18, only low levels of human IL-33 mRNA were detected in this cell type [1]. ECs seem to be the main cell types expressing IL-33 in humans including lung, colon, kidney, liver, skin [22,23], atherosclerotic plaques [33] or adipose tissue [34].

There are some differences in the expression pattern of IL-33 if measured in vivo or in cultured cells in vitro. For example, IL-33 mRNA was detected in cultured arterial smooth muscle cells (SMCs), but not in ECs in vitro by Schmitz et al.[1], and in cultured HUVECs, human saphenous vein ECs (HSVECs), human saphenous vein SMCs (HSVSMCs) and human coronary artery SMCs (HCASMCs) by Miller et al. [35]. In contrast to these in vitro results, only ECs, but not vascular SMCs from arterial blood vessels were shown to constitutively express IL-33 in vivo [22]. Moreover, IL-33 mRNA was described in murine thoracic aorta in normal-fat and high-fat diet-fed mice with increased IL-33 expression in atherosclerotic animals [35]. Our group demonstrated the expression of IL-33 and ST2 protein and mRNA in human atherosclerotic plaques, where nuclear IL-33 was detected in ECs [33]. Increased colocalization of IL-33 with SMCs in atherosclerotic coronary artery sections as compared to arteries without lesions was described in humans [36]. Further studies revealed increased expression of IL-33 and ST2L in vulnerable/high-risk carotid artery plaques, and its expression correlated with the degree of inflammatory cells infiltration [37,38]. Moreover, increased expression of IL-33 was demonstrated in an experimental murine abdominal aortic aneurysm (AAA) model, where it was localized in aortic fibroblasts [39]. Augmented IL-33 expression was also observed in post-guidewire-injured mouse femoral arteries as compared to uninjured artery [36]. IL-33 localization was found in the nuclei of EC in blood vessels of different calibers and also in skin samples from major limb amputations of diabetic critical limb ischemia patients [40].

Moreover, IL-33 expression was upregulated in the aortas of hypertensive mice receiving angiotensin II infusion compared with that of control mice [41], and myocardial pressure overload induces IL-33 in cardiac ECs [42].

IL-33 expression in pooled organ-specific cDNAs and in primary cell cultures demonstrated weakly enhanced IL-33 expression in brain, small intestine, heart and testis compared to very low expressions of IL-33 in skeletal muscle, peripheral blood mononuclear cells (PBMC) and kidney [43]. IL-33 expression was also demonstrated in cultured keratinocytes and skin fibroblasts as well as at lower levels in HUVECs in the same study [43]. We showed a constitutive expression of IL-33 in primary human cardiac fibroblasts, cardiac myocytes and HCASMCs in vitro as well as in human myocardial tissue obtained from explanted hearts of patients undergoing heart transplantation. There, intracellular IL-33 was released during necrosis from those cardiovascular cells [44]. Another study determined IL-33 mRNA in normal and diseased human myocardium as well as IL-33 protein in coronary artery endothelium. Interestingly, IL-33 and ST2L levels were highly correlated in peripheral leukocytes from patients with pressure overload hypertrophy and congestive cardiomyopathy [45].

Constitutive expression of IL-33 protein in its full-length form (31 kDa) was also detected in the cytoplasm of platelets and megakaryocytes [46]. In addition, red blood cells were shown to store and liberate IL-33 upon hemolysis [47].

Historically, caspase-1 was believed to cleave and activate the precursor form of IL-33 to generate an active IL-33 form [1]. However, several studies demonstrated that full-length IL-33_1–270_ is biologically active, and IL-33 is actually inactivated after maturation by caspase-1 [48,49]. Therefore, the regulations of processing for IL-33 and IL-1β are different [50]. In addition, IL-33 is cleaved by apoptosis-associated caspases-3 and caspases-7 into inactive forms [49]. Ohno et al. demonstrated caspase-1-independent, caspase-8-independent and calpain-independent IL-33 release by murine macrophages [51]. Moreover, Cayrol et al. also revealed that full-length IL-33_1–270_ can be released into the extracellular space after injury of ECs [48] and Lüthi et al. proved that endogenous IL-33 is preferentially released during necrosis [49]. Interestingly, chromatin binding reduces IL-33 release during necrosis [31].

In addition to this, full-length human IL-33_1–270_ was shown to be cleaved by cathepsin G and elastase released from neutrophils to generate mature forms IL-33_95–270_, IL-33_99–270_ and IL-33_109–270_ with an intact IL-1-like cytokine domain and a ∼10-fold greater potency to activate ST2L than full-length IL-33_1–270_ [52]. Interestingly, neutrophil proteinase 3 (PR3) enzyme was shown to have dual effects on both activation and termination of IL-33 biological activity [53]. Furthermore, if three purified neutrophil proteases-PR-3, cathepsin G and elastase-were present simultaneously, an inactivation of full-length IL-33 was observed with PR3 being responsible for this effect [54].

A further level of complexity to the biology of IL-33 was added by several reports that indicated an active release of IL-33 by cells that maintain their integrity [55]. One of the examples is adenosine triphosphate (ATP)-mediated secretion of full-length IL-33 in airway epithelial cells during exposure to allergens in mice [56]. Another mechanism includes the secretion of uncleaved IL-33 upon mechanical strain by its translocation to cytoplasmic vesicles from the nucleus in murine fibroblasts in vitro and in vivo in the absence of cellular necrosis [57]. If those mechanisms are also applicable to the cells of the vessel wall is currently unknown.

Thus, IL-33 is believed to be an alarmin that can be released by the damaged cells during necrosis and exerts its biological function in the absence of a proteolytic process or by being processed proteolytically by serine proteases released during inflammation. In rare cases, IL-33 can be also released by living cells under non-lethal stress conditions.

## 4. Regulators of IL-33 Expression in Cells of the Vasculature and Monocytes/Macrophages

ECs were repeatedly shown to be the main source of IL-33 in the vascular wall [22,23]. In this cell type, the induction of IL-33 was described when cultured cells reach confluence and stop proliferating. Simultaneously, IL-33 is lost when ECs begin to migrate [23]. Interestingly, IL-33 expression was not induced when cell cycle progression was inhibited in subconfluent cell cultures. Stimulation of ECs by TNF-α or vascular endothelial growth factor (VEGF) in vitro and subcutaneous injection of both factors in vivo in rats results in a downregulation of vascular IL-33 [36]. The same effect was also observed after incubation of HUVECs with phorbol-12-myristate-13-acetateate [40].

IL-33 levels were augmented in the supernatant of HUVECs after treatment with endosulfan with simultaneous upregulation of RIPK1, RIPK3, mixed lineage kinase domain-like (MLKL), caspase 8 and caspase 3 in another study [58]. NF-κB, PI3K/Akt and Wnt/β-catenin as well as brahma related gene 1 (BRG1, the catalytic component of the mammalian chromatin modeling complex, which is essential for vascular development) pathways were shown to regulate IL-33 transcription in both mouse and human ECs [59,60]. Furthermore, human endothelial IL-33 is activated by adenoviral transduction, which was dependent on the DNA-binding protein meiotic recombination 11 (MRE11), a sensor of DNA damage and the antiviral factor interferon regulatory factor 1 (IRF1) [61].

In human cardiac fibroblasts, cardiac myocytes and HCASMCs, intracellular IL-33 expression was induced by TNF-α, IFN-γ and IL-1β, as was shown by our group [44]. Thrombin stimulated IL-33 expression in human aortic SMCs (HASMCs) and inhibition of IL-33 activity by a neutralizing antibody suppressed thrombin-induced migration of SMCs. Protease-activated receptor 1 (Par1), Gα protein q/11 (Gαq/11), phospholipase Cβ3 (PLCβ3), NF of activated T cells (NFATc1), E2F1 and LIM and cysteine-rich domains protein 1 (LMCD1) were responsible for thrombin-induced IL-33 expression and migration [36]. Altogether, IL-33 expression was required for thrombin-induced proliferation and migration of HASMCs and mouse ASMCs, although a species-specific role of LMCD1 in the expression of IL-33 was found [62]. In primary HASMC, a slight decrease in IL-33 mRNA by IL-22 was demonstrated [63].

Interestingly, the treatment of arteries explanted from atherosclerotic patients with a longevity-associated variant in BPI fold containing family B, member 4 (BPIFB4), increased the release of IL-33 [64].

In human monocytes and mouse peritoneal macrophages, serum amyloid A (SAA), which is known to be an acute-phase protein, induced both IL-33 mRNA expression and nuclear-localized protein. Only little IL-33 protein was measured in the culture medium of human promonocytic THP-1 cells after stimulation with SAA. The induction of IL-33 by SAA was dependent on toll-like receptor 2 (TLR2) signaling and activation of the downstream effector IRF7 [65]. The treatment of macrophages and dendritic cells exposed to oxidized low density lipoprotein (oxLDL) with anti-OX40 ligand (OX40L) increased IL-33 production dose dependently. The same effect was also observed in vivo. When interfering the OX40–OX40L pathway by treatment with anti-OX40L in LDL receptor-deficient mice, an increase in IL-33 expression in the spleen of treated mice compared with control mice in parallel to the regression of atherosclerosis was observed [66].

IL-33 levels were also increased in the cytoplasm of monocytes after stimulation with lipopolysaccharide (LPS) [67]. Sensitivity of RAW264.7 mouse macrophages to LPS with respect to IL-33 production was dependent on cyclic adenosine monophosphate (cAMP) signaling [68]. Distinct expression patterns of IL-33 with low or high dose LPS was also observed in THP-1, where different response levels were dependent on the activation statuses of glycogen sythase kinase 3 (GSK3), Akt, forkhead box O1 (FoxO1) and cAMP response element-binding protein (CREB) [69]. In addition, sterile particles directly promote IL-33 production by peritoneal mouse macrophages, and Bruton’s tyrosine kinase (BTK) signaling specifically augmented the production of IL-33 [70]. Similarly, ultrafine particles generated by combustion processes induced IL-33 release in murine macrophages. Interestingly, in PBMCs, the same effect was observed only in cells obtained from smokers but not from non-smokers [71].

Therefore, IL-33 can be modulated by numerous cellular processes that are activated during tissue stress in different pathological conditions.

## 5. IL-33 in Vascular Pathologies: Protective or Harmful?

### 5.1. In Vitro Effects of IL-33 on Cells of the Vascular Wall and Blood-Born Cells

#### 5.1.1. Effects of IL-33 on ECs

**Figure 1:**
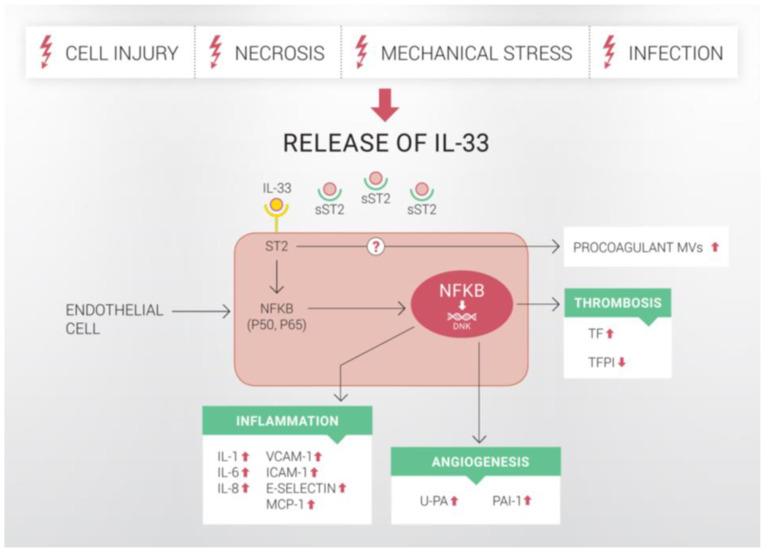
The effects of IL-33 on human ECs. The release of IL-33 occurs following severe tissue damage upon cell injury, necrosis, mechanical stress or infection. When released, IL-33 binds to ST2 receptor on the cell surface as well as to circulating sST2, which negatively regulates IL-33-signalling. Upon binding to ST2 receptor, IL-33 activates several intracellular pathways, including the NF-κB-pathway. It was previously shown that IL-33 induces inflammatory activation of ECs and increases endothelial expression of IL-1, IL-6, IL-8, VCAM-1, ICAM-1, E-selectin and MCP-1. Evidence indicates that IL-33 also increases the expression and activity of u-PA, PAI-1 and TF in human ECs via ST2/NF-κB-pathway. In addition, mRNA and protein expressions of TFPI are reduced. At the same time, IL-33 induces the release of procoagulant MVs from ECs, but the mechanism for this effect remains unclear. In this manner, IL-33 activates human endothelium and promotes a proinflammatory, angiogenic and thrombotic state of human ECs. IL: interleukin; VCAM-1: vascular cell adhesion molecule-1; ICAM-1: intercellular adhesion molecule-1; E-selectin: endothelial selectin; MCP-1: monocyte chemoattractant protein-1; u-PA: urokinase-type plasminogen activator; PAI-1: plasminogen activator inhibitor type-1; TF: tissue factor; TFPI: tissue factor pathway inhibitor; MVs: microvesicles. Upwards arrows indicate upregulation, downwards arrows indicate downregulation.

**Figure 2:**
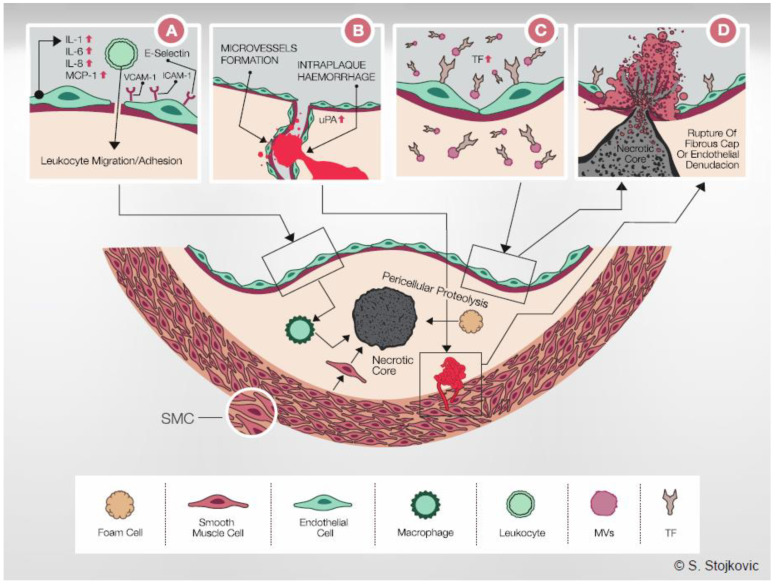
Potential role of IL-33 in the pathogenesis of atherosclerosis. (**A**): IL-33 induces IL-1, IL-6, IL-8, ICAM-1, VCAM-1, E-selectin and MCP-1 in human ECs. This results in the formation of a proinflammatory environment with increased leukocyte adhesion and transmigration. Central panel: Once resident in the arterial intima, monocytes acquire morphological characteristics of macrophages, it undergoes a series of changes that lead ultimately to foam cell formation. Intimal macrophages also secrete u-PA and contribute to SMC migration and intimal thickening. Increased pericellular proteolysis results in degradation of ECM and thinning of the fibrous cap. Later in the development of an atherosclerotic lesion, macrophages, foam cells, SMCs and other cell types present in the plaque become apoptotic and contribute to the necrotic core formation. (**B**): IL-33-induced u-PA expression results in increased migration, tube formation and vessel sprouting of ECs. Furthermore, IL-33 and u-PA are co-expressed in ECs of microvessels within carotid plaques. Formation of microvessels can have nutritive function and stimulate the plaque growth. In addition, fragile microvessels rupture easily and intra-plaque hemorrhage contributes to the formation of an unstable plaque. Thus, by inducing u-PA expression, IL-33 could contribute to both growth and vulnerability of atherosclerotic plaque. (**C**): IL-33 robustly increased TF production and activity in human ECs, as well as the release of procoagulant endothelial MVs. In this manner, IL-33 could result in the formation of a prothrombotic microenvironment surrounding atherosclerotic plaque. Endothelial TF-positive MVs are also present in the necrotic core and increase the thrombogenicity of atherosclerotic plaque. Although IL-33-induced TF expression in ECs alone might not dramatically increase TF content of the entire atherosclerotic plaque, accumulation of TF in ECs can result in apoptosis and contribute to endothelial denudation and to the exposure of the highly prothrombotic core of the atherosclerotic plaque. (**D**): Increased inflammation, pericellular proteolysis, angiogenesis and increased TF production could contribute to growth and destabilization of atherosclerotic plaque, resulting eventually in plaque rupture and thrombosis. IL: interleukin; VCAM-1: vascular cell adhesion molecule-1; ICAM-1: intercellular adhesion molecule-1; E-selectin: endothelial selectin; MCP-1: monocyte chemoattractant protein-1; u-PA: urokinase-type plasminogen activator; TF: tissue factor; MVs: microvesicles.

IL-33 was initially postulated to be an atheroprotective cytokine because its injection reduced atherosclerotic lesions in apolipoprotein E (ApoE) knockout (ApoE^−/−^) mice mainly by the induction of a Th2 response with simultaneous dampening of the Th1 response [35]. However, in human ECs, IL-33 induced inflammatory activation through upregulation of IL-6 and IL-8 [33,72]. IL-33 also promoted adhesion of leukocytes to ECs and increased endothelial production of VCAM-1, ICAM-1, endothelial selectin (E-selectin) and monocyte chemoattractant protein (MCP-1), an early event in the development of endothelial dysfunction and atherogenesis (Figure 1 and Figure 2A) [33]. Furthermore, upon activation with IL-33, ECs also increased expression of chemoattractant ligands CXCL1 and CXCL8, as well as granulocyte macrophage-colony stimulating factor (GM-CSF) and macrophage-CSF [73,74]. In this manner, IL-33 might impact not only adhesion and trans-endothelial migration but also maturation of mononuclear cells. These effects of IL-33 are specific for extracellular IL-33 cytokine and not endogenous IL-33, which does not affect ECs protein expression [32]. The proinflammatory effects of IL-33 in ECs are ST2-receptor mediated, with downstream activation of ERK, JNK/c-Jun, MAPK and NF-κB [33,72,75] and IL-33 targets primarily non-quiescent ECs since ST2 receptor expression is growth-dependent in ECs [72,76].

In addition to proinflammatory activation, IL-33 also increased vascular permeability and promotes angiogenesis and lymphangiogenesis by inducing proliferation, migration and morphologic differentiation of human ECs [16,77]. Simultaneously, nuclear IL-33 expression was either lost during migration and angiogenesis or downregulated during inflammatory activation of ECs [23]. Pericellular proteolysis and cell migration are physiological processes necessary for host defense in inflammation, tissue repair and angiogenesis. Urokinase-type plasminogen activator (u-PA) and plasminogen activator inhibitor type-1 (PAI-1) are key regulators of pericellular proteolysis in ECs [78]. IL-33 altered the proteolytic potential of ECs by inducing u-PA and PAI-1 (Figure 1 and Figure 2B), without any effect on u-PA receptor (uPAR), tissue type PA (t-PA) and matrix metalloproteinase (MMPs)/tissue inhibitors of metalloproteinases (TIMPs) expression. Upregulation of u-PA was necessary for the angiogenic effect of IL-33 in human ECs [79]. In pulmonary ECs, hypoxia induced IL-33 and ST2-receptor expression, and IL-33 initiated vascular remodelling in hypoxic pulmonary hypertension by upregulating hypoxia-inducible factor (HIF)-1α and VEGF expression [80]. Interestingly, IL-33 is an acute vasodilator in isolated small mesenteric arteries, and this effect seems to be endothelium dependent [81].

IL-33 resulted in a prothrombotic state of human ECs [82]. IL-33 induced the production and activity of tissue factor (TF) in human ECs in vitro. IL-33 also increased the release of procoagulant endothelial microvesicles (MVs). In addition to a strong increase in the production and activity of TF, IL-33 downregulated tissue factor pathway inhibitor (TFPI) expression in ECs in vitro. The IL-33-induced increase in TF in human ECs had an impact on the coagulation time of human whole blood and plasma ex vivo [79]. Thus, IL-33 might adversely affect the hemostatic balance in ECs and shift it towards a prothrombotic state (Figure 1 and Figure 2C). In addition, IL-33-induced expression of adhesion molecules on ECs could result in increased binding and activation of leukocytes and leukocyte-derived MVs to ECs [33,83].

#### 5.1.2. Effects of IL-33 in Monocytes/Macrophages

In regard to thrombosis, the effects of IL-33 on monocytes might be of even greater interest, since monocytes and monocyte-derived MVs are one of the main sources of circulating TF and contribute to the formation of a prothrombotic environment in patients with cardiovascular disease through the propagation of coagulation upon plaque rupture [84,85,86]. Monocytes constitutively express IL-33 and increased its expression in acute and chronic inflammation. Thus, IL-33 can be released upon cellular damage or by necrotic cells [67]. Monocytes also express ST2 receptor with distinct ST2 expression in monocyte subsets, with intermediate and non-classical monocytes having much higher expressions levels of the ST2 receptor as compared to classical monocytes [87]. Therefore, it is not surprising that IL-33 induced TF expression and activity predominantly in intermediate monocytes, as well as the release of TF-positive monocyte-derived MVs [87]. Thus, through its effects on monocytes and ECs, IL-33 could promote both inflammation and the formation of a prothrombotic state characteristic for patients with cardiovascular disease.

Macrophages also constitutively express ST2 receptor. However, the effects of IL-33 on macrophage activation are controversial. IL-33 resulted in a proinflammatory M1 macrophage response and cytokine release [88], and in the setting of acute inflammation, IL-33 enhanced macrophage activation by LPS [89]. In contrast, one study showed that IL-33 induced alternative macrophage activation in mice [90]. In macrophage-derived foam cells, IL-33 reduced cellular cholesterol level and upregulated IL-10 expression [5]. IL-33 also inhibited foam cell formation via a decrease in oxLDL uptake and an increase in cholesterol efflux [91]. Furthermore, IL-33 downregulated the expression of a disintegrin and metalloproteinase with thrombospondin motifs (ADAMTS) family of metalloproteases via ERK1/2, JNK and PI3K-AKT signaling in macrophages [92]. In contrast to ECs, IL-33 reduced MCP-1 and ICAM-1 expression in macrophages [93]. However, IL-33 regulated the recruitment of monocytes and granulocytes via B1 cell release of the chemokines MCP-1 and macrophage inflammatory protein-1 alpha and also induced VEGF and GM-CSF in these cells [94]. Furthermore, IL-33 regulates neutrophil recruitment and mast cell activation [95]. These results suggest a cell-type and disease-type specific action of IL-33, which needs to be further delineated in future studies.

Both IL-33 and ST2 were previously detected in human atherosclerotic plaques [33,79,82]. Dysfunction of ECs with excessive u-PA activity and pathological pericellular proteolysis is characteristic for growth and subsequent vulnerability of atherosclerotic plaques [96,97]. Neovascularization is one of the most important mechanisms, which contributes to the destabilization and rupture of atherosclerotic plaques [97]. IL-33 and u-PA were co-expressed in ECs of microvessels in symptomatic and asymptomatic carotid atherosclerotic plaques, and their levels show strong positive correlation [79]. Newly formed microvessels within the plaque can nourish the plaque and contribute to its growth [98]. Such microvessels are known to be fragile and rupture easily (Figure 2B). Intra-plaque hemorrhage and thrombosis cause further narrowing of the lumen of a diseased artery and plaque destabilization [98]. IL-33 expression was also co-localized with TF in ECs of microvessels in atherosclerotic plaques and especially at the site of in situ thrombus formation within symptomatic carotid atherosclerotic plaques [82]. The levels of IL-33 and TF also showed strong positive correlation in these lesions [82]. Thrombin induces IL-33 in SMCs, and its expression is necessary for migration and injury-induced neointimal growth [36]. Finally, elevated levels of circulating IL-33 are associated with adverse atherothrombotic events in patients with coronary artery disease and carotid stenosis [99,100,101].

Therefore, described in murine atherosclerosis models as well as in macrophages as an atheroprotective cytokine, extracellular IL-33 induces proinflammatory, prothrombotic and proangiogenic activation of human ECs, which are processes known to be involved in development and progression of atherosclerosis (Figure 2D).

### 5.2. In Vivo Effects of IL-33 in Models of Vascular Diseases

The first report, which designated IL-33 as a member of the IL-1 superfamily [1], described that injection of IL-33 (0.4 or 4 μg daily) in mice for a short time (up to 7 days) induces splenomegaly, eosinophilia and serum IgE as well as pathological changes in the lung and the digestive tract. IL-33 injection also resulted in the production of Th2-associated cytokines [77]. IL-33 injections also induce eosinophilia and splenomegaly in lean and obese mice although the degree of eosinophilia was higher in lean mice [81]. Eosinophilic number was also increased in perivascular adipose tissue after IL-33 injection [81]. The comparison of full-length (not proteolytically processed) mouse and mature mouse forms of IL-33 demonstrated that both isoforms resulted in pulmonary infiltration of lymphocytes and neutrophils, whereas the mature IL-33 form also caused pulmonary eosinophilia, goblet cell hyperplasia and increased expression of Th2 cytokines and MCP-1 [102].

Miller et al. investigated possible effects of IL-33 in murine atherosclerosis and demonstrated that injection of a high dose of IL-33 (1 μg/injection twice per week) for 6 weeks into atherosclerosis-prone ApoE^−/−^ mice reduced the development of atherosclerotic lesions in the aortic sinus [35]. In parallel, IL-33 injection reduced the percentages of F4/80^+^ macrophages or T lymphocytes infiltrating atherosclerotic lesions with simultaneous increase in the Th2 cytokines IL-4, IL-5 and IL-13 as well as oxLDL-specific antibodies in serum. However, IL-33 did not affect collagen content or SMCs in the plaques [35]. In contrast to those results, the deficiency in IL-33/ST2 signaling did not affect atherosclerosis severity in double knock-out IL-33^−/−^ApoE^−/−^ and ST2^−/−^ApoE^−/−^ mice [103]. One explanation for this discrepancy could be the fact that the systemic administration of IL-33 induces Th2 responses, which might result in a different outcome in the setting of atherosclerosis as compared to physiological amounts of endogenous IL-33 [35,103].

Li and colleagues investigated the role of IL-33 in murine models of AAA. The authors found that overexpression of IL-33 decreased AAA size as well as infiltration of T-cells and macrophages with a shift to increased M2 macrophage polarization. The protective action of exogenous IL-33 on AAA growth was also dependent on regulatory T cells [39]. Exogenous IL-33 also antagonized angiotensin II and phenylephrine-induced cardiomyocyte hypertrophy. Moreover, the injection of IL-33 reduced hypertrophy and improved survival in a murine transverse aortic constriction (TAC) model. Thus, IL-33/ST2 signaling was proposed to be a cardioprotective fibroblast-cardiomyocyte paracrine system [104]. In mice, the consequence of myocardial pressure overload during TAC was an increase in systemic endothelial-derived IL-33. Conditional endothelial-specific deletion of IL-33 aggravated pressure overload-induced hypertrophy in that model. The authors concluded that EC-secreted IL-33 is specifically important for transformation of myocardial pressure overload into a systemic inflammatory state [42].

In contrast, injury-induced neointimal growth was shown to require IL-33 expression, and IL-33 neutralizing antibodies reduced SMC migration and neointimal growth in a murine femoral artery guidewire injury model in vivo [36]. In a mouse model of hypoxia-induced pulmonary hypertension exogenous IL-33 aggravated pulmonary hypertension that was evident by elevation of the mean right ventricular systolic pressure and right ventricular hypertrophy index [80]. However, a beneficial effect of IL-33 on blood pressure was reported in IL-33 treated wild-type lean and obese mice [81].

Therefore, IL-33 can produce both protective and harmful effects in experimental models of vascular pathologies depending on experimental conditions, type and dose of administration or method of modulation such as, e.g., exogenous administration, conditional deletion and whole-body knock out.

## 6. Summary and Perspectives

IL-33 is both an extracellular cytokine in case of its release from cells exposed to stress or damage as well as a nuclear-located factor with the ability to regulate transcription. Although transcriptional repressor properties were described for IL-33 as a heterochromatin-associated nuclear factor, the certain role of nuclear IL-33 is still not completely understood. IL-33 can be released not only during necrosis but also by living cells under non-lethal stress conditions, although the data on IL-33 release by living vascular cells are limited. In ST2-expressing cells, IL-33 activates multiple signaling pathways. What is particularly interesting is the activating function of IL-33 in human ECs where it induces proinflammatory, proangiogenic and prothrombotic milieu. Simultaneously, ECs seem to be the main source of extracellular IL-33. In contrast to its postulated role as atheroprotective cytokine when injected into mice, IL-33 deficiency had no influence on atherosclerosis development in double knock-out IL-33^−/−^ApoE^−/−^ mice. It would be extremely important to test atherosclerosis development in murine models with conditional deletions of IL-33 and ST2, e.g., in ECs. It can also be imagined that the IL-33/ST2 axis might play different roles in early development of atherosclerosis vs. destabilization of chronic, already established atherosclerotic lesions. Such questions need to be answered before a possible therapeutic modulation of IL-33 can be considered for vascular pathologies.

## Abbreviations

AAA	abdominal aortic aneurysm
ADAMTS	a disintegrin and metalloproteinase with thrombospondin motifs
ApoE	apolipoprotein E
ATP	adenosine triphosphate
BPIFB4	BPI fold containing family B, member 4
BRG1	brahma-related gene 1
BTK	Bruton’s tyrosine kinase
cAMP	cyclic adenosine monophosphate
CBM	chromatin-binding motif
CREB	cAMP response element-binding protein
ECs	endothelial cells
eNOS	endothelial nitric oxide synthase
Erk	extracellular signal-regulated kinase
FoxO1	forkhead box O1
Gαq/11	Gα protein q/11
GM-CSF	granulocyte macrophage-colony stimulating factor
GSK3	glycogen sythase kinase 3
HASMCs	human aortic SMCs
HCASMCs	human coronary artery SMCs
HIF	hypoxia-inducible factor
HEVECs	high endothelial venules endothelial cells
HSVECs	human saphenous vein ECs
HSVSMCs	human saphenous vein SMCs
HTH	Helix-Turn-Helix
HUVECs	human umbilical vein endothelial cells
ICAM	intercellular adhesion molecule-1
Ig	immunoglobulin
IκBα	inhibitor of κBα
IL	interleukin
IL1RAcP	IL-1R accessory protein
IL1RL1	IL-1-receptor-like 1
Inpp5d	Inositol polyphosphate-5-phosphatase D
IRAK	IL-1R-associated kinase
IRF1	Interferon Regulatory Factor 1
JAK	Janus kinase
JNK	c-Jun N-terminal kinases
kDA	kilodalton
LDL	low density lipoprotein
LMCD1	LIM and cysteine-rich domains protein 1
LPS	lipopolysaccharide
MAPK	mitogen-activated protein kinases
miRs	microRNAs
MCP-1	monocyte chemoattractant protein
MLKL	mixed lineage kinase domain-like
MMP	matrix metalloproteinase
MVs	microvesicles
MyD88	myeloid differentiation factor 88
MYPT1	myosin phosphatase target subunit 1
NAD	nicotinamide adenine dinucleotide
NF	nuclear factor
NFATc1	NF of activated T cells
NF-HEV	NF from human high endothelial venules
NF-kB	nuclear factor-kappa B
oxLDL	oxidized low-density lipoprotein
PAI-1	plasminogen activator inhibitor type-1
Par1	protease-activated receptor 1
PBMC	peripheral blood mononuclear cells
PI3K	phosphoinositide-3-kinase
PLCβ3	phospholipase Cβ3
PR3	proteinase 3
PTPN12	Protein Tyrosine Phosphatase Non-Receptor Type 12
RIPK	receptor-interacting protein kinase
SAA	serum amyloid A
SIGIRR	single Ig IL-1R-related molecule
siRNA	silencing RNA
SMC	smooth muscle cell
sST2	soluble ST2
ST2	suppression of tumorigenicity 2
ST2L	membrane-bound ST2
SYK	spleen tyrosine kinase
TAC	transverse aortic constriction
TF	tissue factor
TFPI	tissue factor pathway inhibitor
Th	T helper
TIMP	tissue inhibitor of metalloproteinases
TIR	toll/IL-1 receptor
TLR2	toll-like receptor 2
TMED1	transmembrane emp24 domain-containing protein 1
TNF	tumor necrosis factor
t-PA	tissue-type plasminogen activator
TRAF6	TNF-α receptor-associated factor 6
u-PA	urokinase-type plasminogen activator
uPAR	u-PA receptor
VCAM-1	vascular cell adhesion molecule-1
VEGF	vascular endothelial growth factor
